# Complexity of gastroschisis predicts outcome: epidemiology and experience in an Australian tertiary centre

**DOI:** 10.1186/s12884-018-1867-1

**Published:** 2018-06-11

**Authors:** Sarah J. Melov, Irene Tsang, Ralph Cohen, Nadia Badawi, Karen Walker, Soundappan S. V. Soundappan, Thushari I. Alahakoon

**Affiliations:** 1 Westmead Institute for Maternal and Fetal Medicine, Women’s and Newborn Health, Westmead Hospital, Research & Education Network Building, Hawkesbury Rd, PO Box 533, Westmead, NSW 2145 Australia; 2 Department of Paediatric Surgery, The Children’s Hospital at Westmead, The Sydney Children’s Hospital Network, Hawkesbury Rd, Westmead, NSW 2145 Australia; 3 Grace Centre for Newborn Intensive Care, The Children’s Hospital at Westmead, The Sydney Children’s Hospital Network, Hawkesbury Rd, Westmead, Wentworthville, NSW 2145 Australia; 4 The University of Sydney, Sydney, NSW Australia; 5 Cerebral Palsy Alliance Research Institute, Sydney, NSW Australia

**Keywords:** Gastroschisis, Antenatal diagnosis, Outcome, Incidence, Stillbirth, Length of stay, Congenital anomaly

## Abstract

**Background:**

Gastroschisis is a congenital anomaly of the fetal abdominal wall, usually to the right side of umbilical insertion. It is often detected by routine antenatal ultrasound. Significant maternal and pediatric resources are utilised in the care of women and infants with gastroschisis. Increasing rates of gastroschisis worldwide have led institutions to review local data and investigate outcomes. A collaborative project was developed to review local epidemiology and investigate antenatal and neonatal factors influencing hospital length of stay (LOS) and total parental nutrition (TPN) in infants born with gastroschisis.

**Methods:**

We performed a five-year review of infants born with gastroschisis (2011–2015) at a major Australian centre. Complex gastroschisis was defined as involvement of stenosis, atresia, ischemia, volvulus or perforation and closed or vanishing gastroschisis. We extracted data from files and databases at the two participating hospitals, a major maternal fetal medicine centre and the affiliated children’s hospital.

**Results:**

There were 56 infants antenatally diagnosed with gastroschisis with no terminations, one stillbirth (2%) and one infant with ‘vanishing’ gastroschisis. The mean maternal age was 23.9 years (range, 15–39 years). The mean gestation at delivery was 36 weeks (range, 25–39^+ 3^ weeks). Of the 55 neonates who received surgical management, 62% had primary closure. The median LOS was 33 (IQR, 23–45) days and the median duration of TPN was 26 (IQR, 17–36) days. Longer days on TPN (median 35 vs 16 days, *P* = 0.03) was associated with antenatal finding of multiple dilated bowel loops. Postnatal diagnosis of complex gastroschisis was made in 16% of cases and was associated with both longer LOS (median 89 vs 30 days, *P* = 0.003) and days on TPN (median 46 vs 21 days, *P* = 0.009).

**Conclusion:**

Complex gastroschisis was associated with greater days on TPN and LOS. We found no late-gestation stillbirths and a low overall rate of 1.8%, suggesting the risk for stillbirth associated with gastroschisis is lower than previously documented. This information may assist counselling families. Improved data collection worldwide may reveal causative factors and enable antenatal outcome predictors.

## Background

Gastroschisis is a congenital defect of the fetal abdominal wall, resulting in extrusion of abdominal contents. It is usually diagnosed in the antenatal period [1] and requires early neonatal surgical intervention. Extensive resources of maternal fetal medicine, neonatology and pediatric surgery are used in antenatal diagnosis, neonatal management and treatment of infants with gastroschisis. Published literature has documented a 14% termination rate with early diagnosis [1].

Rising rates of gastroschisis have been reported worldwide, with a well-recognized higher prevalence in younger women [2, 3]. From an incidence of 0.06–0.8 per 10,000 in the 1960s, [4–6] gastroschisis has become more prevalent over the last few decades to its current rates of 4.5–5.13 per 10,000 pregnancies [7–9]. Global variations in incidence have been reported, with 10.9 per 10,000 [10] in Greenland and 29.9 per 10,000 in Mexico [11]. Ethnic variations in incidence [9, 12] and evidence of regional or geospatial clustering have also been described [13, 14]. Controversy and conflicting reports have existed about the influence of terminations [15] and misdiagnosis or incomplete data capture [16] on reporting of the incidence of gastroschisis.

The reason for a higher prevalence of gastroschisis in the babies of a younger maternal age group is not clear. Studies investigating its association with factors such as maternal drug use, smoking, [7, 17] nutritional factors, paternal age, [6] maternal infection, [18] pesticide use [19] and other environmental agents [20] have not been conclusive, except that an association with smoking has been consistent [21].

Many studies of gastroschisis have focused on finding definitive antenatal predictors for adverse events, but no clear prognostic indicators have been found to assist with management or counselling of women and their families. A recent meta-analysis of 26 studies had inconclusive results, stating that only intra-abdominal bowel dilatation (IABD), stomach enlargement and polyhydramnios may be associated with more adverse outcomes [22].

Controversy exists regarding best practice for monitoring and delivering babies with gastroschisis. “Matting” a type of bowel injury characterized by varying degrees of thickened bowel wall, rigidity, adherent dilated bowel loops and discoloration is thought to be associated with poorer outcome [23]. It has been suggested that delivery at earlier gestation reduces bowel matting. However, the aetiology of matting is poorly understood and longer exposure to amniotic fluid has not been associated with increased bowel matting. Youssef [24] found a 3.6% decrease in severe matting with every extra week a fetus was in utero. Planned delivery prior to 36 weeks of completed gestation does not seem to confer any short- or long-term outcome advantages but may contribute to adverse outcomes [25, 26].

Westmead Hospital (WH) and The Children’s Hospital at Westmead (CHW) are affiliated tertiary referral centres, and one of only three facilities in New South Wales, Australia that provide a collaborative tertiary pediatric surgical service for pregnancies complicated by gastroschisis. The state of New South Wales (NSW) has mandatory reporting to the NSW Register of Congenital Conditions. Aggregate data on gastroschisis was provided by the NSW Centre for Epidemiology and Evidence, Ministry of Health data for the last ten-year period available (2004–2013) in the NSW Register of Congenital Conditions database. This data reveals Westmead Hospital cared for more gastroschisis cases than any other hospital in the state (*n* = 78, 35.5%).

The primary aim in our study at WH and CHW, was to determine predictors for hospital length of stay (LOS) and days on total parental nutrition (TPN). Our secondary objectives were to review epidemiology and uncover other predictors for infant outcome to discharge, generating important local data to inform service planning for the leading centres of care for gastroschisis in NSW, Australia.

## Methods

We conducted a five-year retrospective review of all neonates antenatally diagnosed with gastroschisis and born at WH during the five-year period from January 2011 to December 2015. Patients were identified using existing databases at WH and CHW. Antenatal data were extracted from the WH obstetric and ultrasound database. Neonatal data were retrieved from the CHW Grace Centre for Newborn Intensive Care database and the surgical database. We reviewed individual patient notes for all cases.

Data recorded included maternal demographic data, antenatal risk factors, delivery details and postnatal management aspects including LOS, TPN, categorisation into complex or simple gastroschisis, primary or secondary closure and infection, with data collected until patient discharge. Complex gastroschisis was defined as additional intestinal morbidity at first postnatal surgical evaluation of any of the following: stenosis, atresia, ischemia, volvulus, perforation [27] and closed or vanishing gastroschisis. Bowel dilation in the fetus was defined as any dilation of the bowel greater than 8 mm [28]. Small for gestational age was defined as estimated fetal weight < 10th centile, with polyhydramnios and oligohydramnios defined by reference amniotic fluid index nomograms for gestation used in our ultrasound practice [29].

Time on TPN was the duration until full enteral feeding was achieved.

Antenatal care at our institution for women with a gastroschisis baby includes care with the high risk maternal fetal medicine clinic, a named maternal fetal medicine specialist consultant and named caseload midwife from booking for continuity of care. Surveillance for gastroschisis includes ultrasound monitoring every 2 weeks from 28 weeks and a fetal cardiotocography (CTG) three times per week from 32 weeks.

### Statistical analyses

We analysed the associations between the primary outcomes of LOS and time on TPN and the clinical or demographic factors of country of mother’s birth, insurance status, gestation at diagnosis, gestation at first maternal fetal medicine consultation, day and time of delivery, gestation at birth, fetal gender, mode of delivery, as well as if they had a surgical primary or delayed closure.

Statistical analysis was performed using SAS 9.4. Continuous variables are presented as median and interquartile range if skewed and mean (standard deviation) or range otherwise. Categorical variables were described by frequencies and percentages.

Associations between clinical and demographic factors and study outcomes were examined using chi-square tests, logistic regression, Spearman correlation and the Wilcoxon rank-sum test. There was no adjustment made for multiple statistical comparisons. All statistical tests were performed at a 2-sided level, where *P* < 0.05 is considered statistically significant.

## Results

There were 56 babies with gastroschisis identified antenatally in the five-year period, born at WH. One fetal death at 25 weeks was reported with a localised stricture of an amniotic band at the fetal end of the umbilical cord and was excluded from outcome analysis. The only fetus with a concurrent anomaly had mild hydronephosis and was included in outcome analysis.

There were 55 babies with gastroschisis in the five-year period that were analysed for neonatal outcome of LOS and days on TPN. One mother gave birth to two infants with gastroschisis over consecutive study years. Another mother gave birth to a second infant with gastroschisis in the year after the study period. No postnatal diagnosis of gastroschisis was made and there were no terminations of a pregnancy in the study period of a fetus diagnosed with gastroschisis.

Most patients identified were from outside the local hospital district, with 30% (*n* = 17) from within the local tertiary referral hospital district area and three from New Caledonia. Of the 45 babies of known gestation at diagnosis, 60% (*n* = 27) were diagnosed before or at 14 weeks, with 25 (56%) women diagnosed at their nuchal translucency screening ultrasound. The mean gestation at primary diagnosis was 15 weeks (range, 11–22 weeks).

The mean maternal age was 23.9 years (range, 15–39 years). Most women (64.3%) recorded a body mass index in the normal range (18.5 - < 25 kg/m^2^) at first booking-in visit (Table 1). All patients had spontaneous conception and there were two twin pregnancies recorded (one dichorionic diamniotic pregnancy and one monochorionic diamniotic pregnancy). One patient disclosed recent cannabis use and one polysubstance use, with 27% of women reporting current cigarette smoking (Table 1). Genitourinary infections have been proposed to be associated with gastroschisis [30], in our study 17.9% (*n* = 10) of all women had a history of recurrent urinary infections. Table 1 reports other medical conditions that were disclosed by the 56 women with ≥10 frequency.

**Table 1:** Demographic data for mothers of babies diagnosed with gastroschisis and born at Westmead Hospital (*n* = 56), compared with data for New South Wales, 2011–2015

Characteristic	Westmead Hospital, 2011–2015, (*n*)%	All NSW, 2011–2015,^*^ mean % (range)
Maternal age, years		
≤ 20	13(23.2%)	2.9% (2.5–3.2%)
20–24	22(39.3%)	12.6% (12.0–13.1%)
25–29	13 (23.2%)	27.2% (26.8–27.7%)
≥ 30	8 (14.3%)	57.3% (56–58.7%)
Aboriginal	7 (12.5%)	3.6% (3.1–4.0%)
Body mass index^3^		N/A
< 18.5	2 (3.6%)	
18.5 to < 25	36 (64.3%)	
25 to < 30	14 (25%)	
≥ 30	4 (7.1%)	
Smoking		
Yes	15 (26.8%)	9.9% (8.9–11.1%)
No	41 (73.2%)	90.1% (88.9–91.1%)
Medical history		N/A
Asthma	19 (33.9%)	
Anxiety disorder	11 (19.6%)	
Eczema	10 (17.9%)	
Genital herpes	3 (5.4%)	
Chlamydia	2 (3.6%)	
Recurrent UTI^a^	10 (17.9%)	
Parity		
First birth	37 (66.1%)	43.9% (43.4–44.2%)
≥ Second birth	19 (33.9%)	56.1% (55.8–56.6%)
Consanguineous		N/A
No	55 (98.2%)	
Yes	1 (1.8%)	
Country of birth		
Australia	41 (73.2%)	64.8% (63.3–66.4%)
Non-Australian	15 (26.8%)	35.2% (33.6–36.7%)

^a^Urinary Tract Infections

*N/A* not available, *NSW* New South Wales, *Source: Centre for Epidemiology and Evidence; NSW Mothers and Babies 2015. Sydney, NSW Ministry of Health, 2016

Thirteen percent of women (*n* = 7) identified as Aboriginal (Table 1) and none identified as Torres Strait Islander. Other nationalities represented in the cohort were New Caledonia (*n* = 3), New Zealand (*n* = 3), the Philippines (*n* = 2) and Lebanon (*n* = 2). There were no women born in an East Asian or South Asian country group (United Nations geoscheme) in our cohort, these are the two most common groups (Table 2) in our local area of the Western Sydney Local Health District (WSLHD). In New South Wales maternity databases only have country of birth and not ethnicity as a data collection point.

**Table 2:** Maternal COB: Babies with Gastroschisis from addresses in WSLHD only, 2011–2015

Variable	Babies born with gastroschisis WSLHD address, *n* = 17	All WSLHD births, *n* = 49,647
Gastroschisis	3.4 per 10,000	–
Australian-born mother	10 (58.8%)	17,782 (35.8%)
Non-Australian-born mother	7 (41.2%)	31,865 (64.2%)
^a^Southern Asia	0 (0.0%)	13,261 (26.7%)
East Asia	0 (0.0%)	5516 (11.1%)

^a^Most common non-Australian born group in WSLHD

*WSLHD* Western Sydney Local Health District, *COB* Country of Birth

Of the 30 women who attended their nuchal translucency combined first trimester screening, 93% of results (*n* = 28) were recorded as low risk. None of the high risk results proved to have karyotype abnormalities. At the 18–22-week ultrasound, there were 28 reports of intra-abdominal dilated bowel loops, with 21% (*n* = 6) noting the presence of multiple dilated bowel loops on scan images at this gestation. There were 29 cases that reported to show fetal intra-abdominal dilated bowel loops, of these 55% (*n* = 16) documented multiple dilated bowel loops present on their ultrasound. Significant bowel dilation > 18 mm [31] was found in 35% (*n* = 19) and was not associated with greater days on TPN or LOS. Stomach enlargement was not documented as a significant finding in any of our antenatal ultrasounds.

Polyhydramnios was recognised in seven patients and oligohydramnios in four. Small for gestational age was predicted on ultrasound in 40% of fetuses (*n* = 22) after 25 weeks. An antenatal review by a pediatric surgeon was recorded in 66% of cases at a mean gestational age of 30 weeks (range, 14–38 weeks).

Table 3 details birth information. The mean gestation at delivery was 36 weeks (range, 25–39^+ 3^ weeks), with a 1:1.2 infant male:female ratio. Most births (55.4%) occurred out of normal working hours (Table 4). The median LOS was 1.3 h (IQR, 1.1–2.2 h) in the Westmead neonatal intensive care unit for initial stabilisation prior to transfer via an enclosed walkway to the adjacent Children’s Hospital. A total of 62% of babies had primary closure of the defect (see Table 5 for a summary of surgical details). The median LOS was 33 days (IQR, 23–45 days) with the median duration of TPN 26 days (IQR, 17–36 days) (Table 5).

**Table 3:** Data for babies diagnosed with gastroschisis in WSLHD, compared with all NSW births, 2011–2015

Variable	Diagnosed with gastroschisis, *n* = 56	All NSW births,^*^ mean % (range)
Pregnancy outcome, *n* (%)		
Live birth	55 (98.2%)	99.14% (99.1–99.2%)
Stillbirth	1 (1.8%)	0.58% (0.5–0.6%)
Neonatal death	0	0.22% (0.2–0.3%)
Weeks’ gestation at birth, *n* (%)		
< 31	2 (3.6%)	0.72% (0.7–0.8%)
32–36	34 (60.7%)	6.22% (6.0–6.4%)
37–41	20 (35.7%)	91.82% (91.7–91.9%)
Labour induced	23 (41.0%)	28.7% (26.6–30.5%)
Mode of delivery, *n* (%)		
Vaginal	24 (42.8%)	56.6% (56–57.1%)
Instrumental	2 (3.6%)	11.3% (11.1–11.4%)
Caesarean section	30 (53.6%)	31.7% (31.1–32.4%)
Sex of baby, *n* (%)		
Male	25 (44.6%)	51.3% (51.3–51.4%)
Female	31 (55.4%)	48.7% (48.5–48.7%)
Apgar score at 5 min ≥ 7, *n* (%)	52 (92.9%)	97.5% (97.4–97.6%)
Live birth median weight, (range) g	2400 (1300–3860)	N/A

*NSW* New South Wales, *N/A* not available, *Source: Centre for Epidemiology and Evidence; NSW Mothers and Babies 2015. Sydney, NSW Ministry of Health, 2016

**Table 4:** Variable association with primary outcome LOS, time on any TPN

	Total *n* = 55	TPN		LOS	
Variable	n *(%)*	Median days Rho (r)	*P* value	Median days Rho (r)	*P* value
Country of Birth^†^					
Australia	40 (73)	26	0.73	33	0.51
Other	15(27)	22	–	30	–
Health insurance^†^					
No private	46 (84)	27	0.87	34	0.29
Private	9 (16)	19	–	27	–
Gestation^∞^ at diagnosis *n* = 44	(80)	*r* = 0.15	0.32	*r* = 0.07	0.63
Gestation^∞^ at MFM review *n* = 46	(84)	*r* = 0.07	0.64	*r* = −0.01	0.94
Ultrasound †					
Fetal bowel dilation > 18 mm	19 (35)	31	0.17	37	0.27
Fetal bowel dilation < 18 mm	35 (65)	21	–	30	–
Polyhydramnios	7 (13)	22	0.5	26	0.20
No polyhydramnios	47 (87)	28	–	35	–
Multiple dilated bowel loops	16 (30)	35	0.03	38	0.06
No Multiple dilated loops	38 (70)	16	–	27	–
Bowel wall > 3 mm	4 (7)	24	0.78	28	0.40
Bowel wall < 3 mm	50 (93)	26	–	34	–
Birth time/day^†^					
Mon-Fri 8 am-8 pm	24 (44)	29	0.44	37	0.18
Mon-Fri 8 pm-8 am	17 (31)	28	–	42	–
Sat-Sun 8 am-8 pm	6 (11)	24	–	31	–
Sat-Sun 8 pm-8 am	8 (15)	19	–	24	–
Gestation at birth^∞^ n = 55	(100)	*r* = −0.11	0.42	*r* = −0.15	0.27
Caesarean birth^†^	30 (56)	27	0.90	35	0.35
Vaginal birth	24 (44)	23	–	30	–
Female gender^†^	31 (56)	22	0.91	33	0.73
Male gender	24 (44)	27	–	34	–
Birth weight^∞^ n = 55	(100)	*r* = −0.13	0.36	*r* = −0.17	0.21
Simple gastroschisis^†^	46 (84)	21	0.009	30	0.003
Complex gastroschisis	9 (16)	46	–	89	–
Primary closure^†^	34 (62)	23	0.60	30	0.19
Secondary Closure	21 (38)	28	–	37	–

*P* value is based as indicated: Spearman’s correlation coefficient∞ with Rho(r) reported in table

Wilcoxon rank-sum test † with median reported in table

Some missing data therefore total may not equal 55

*LOS* length of stay, *TPN* total parenteral nutrition, *US* Ultrasound, *MFM* Maternal Fetal Medicine

**Table 5:** Characteristics of neonatal gastroschisis, Westmead Hospital/CHW, 2011–2015, *n* = 55

Characteristic	*n*	Median (IQR)
Total LOS, both Hospital^a^, *days*	55	33 (23–45)
LOS, Complex gastroschisis, *days*	9	89 (57–147)
LOS, Simple gastroschisis, *days*	46	30 (23–39)
Duration of TPN, *days*	55	26 (17–36)
TPN, Complex gastroschisis, *days*	9	46 (34–187)
PN, Simple gastroschisis, *days*	46	21 (17–31)
Duration of tube feeding, *days*	51	20 (13–32)
Duration of CVL, *days*	55	28 (18–43)
Age at primary surgery, *hours*	29	5 (5–7)
Surgical findings^b^ *cases* (%)		
Bowel perforation	2 (3.6)	
Bladder herniated	4 (7.3)	
Presence of peel	22 (40.0)	
Presence of matting	7 (12.7)	
Bowel ischemia	3 (5.5)	
Intestinal atresia present	7 (12.7)	
Adhesions	2 (3.6)	
Primary repair surgery,	34 (61.8)	
Primary repair with skin	3/34 (8.8)	
Primary repair skin/muscle/fascia	28/34 (82.4)	
Use of patch for closure	3/34 (8.8)	
Delayed closure surgery	21 (38.2)	
Delayed surgery final closure, *Mean ± SD, days*	5.5 ± 2.5, range, 1–11	

^a^Westmead Hospital, Neonatal Intensive Care and Grace Centre for Newborn Intensive Care: The Children’s Hospital Westmead (CHW)

^b^Surgical finding: complications do not add up to 100% due to overlapping

*LOS* length of stay, *IQR* interquartile range, *TPN* total parenteral nutrition, *CVL* central venous line

No independent factor had a significant impact on both LOS and duration of TPN except postnatal diagnosis of complex gastroschisis, however the finding at any gestation of multiple dilated bowel loops was associated with more days on TPN (*P* = 0.03) (Table 4).

**Fig. 1:**
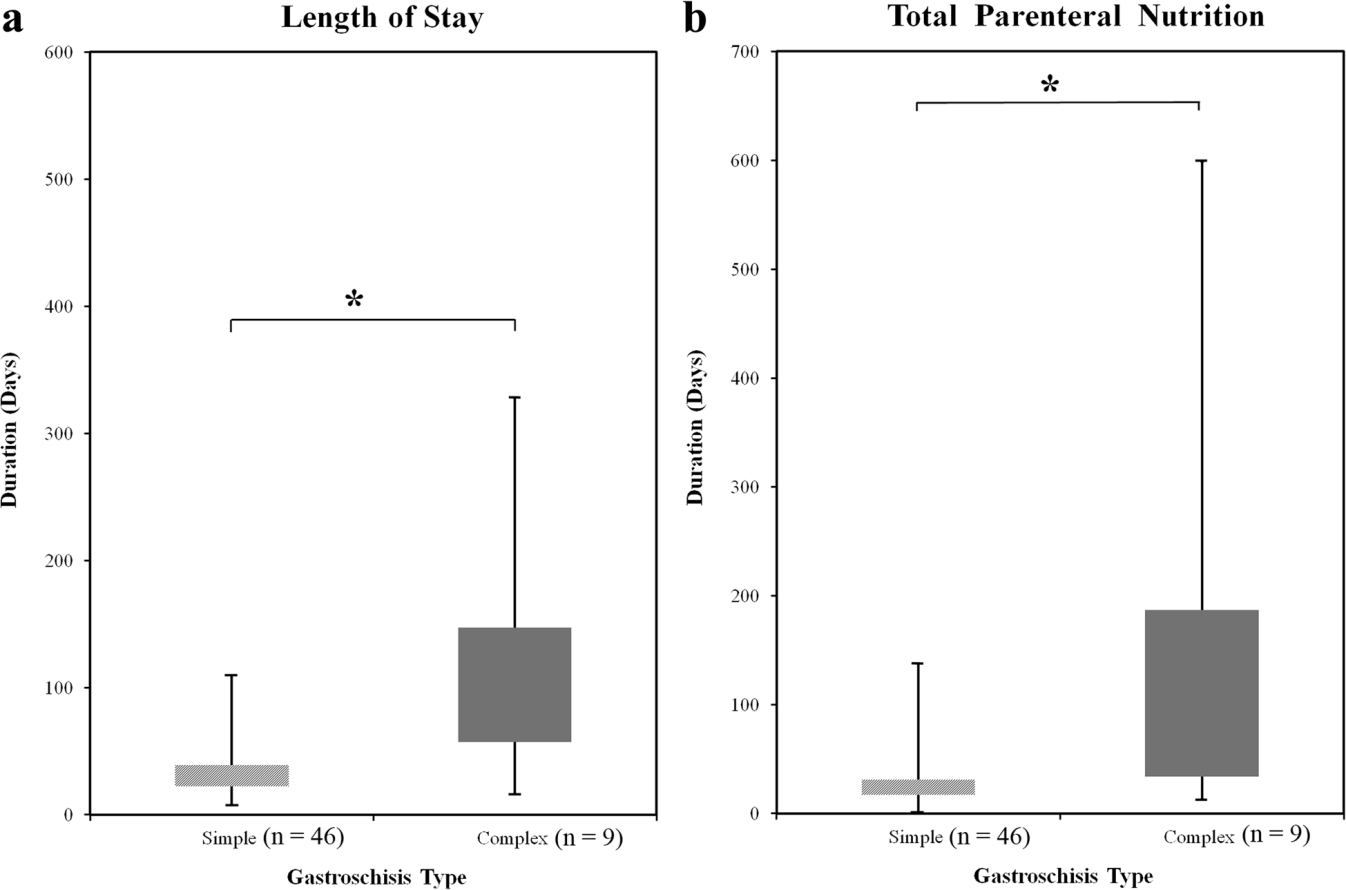
The primary outcomes length of stay in hospital (LOS) and days on total parenteral nutrition (TPN) for the live-born babies with simple and complex gastroschisis. **a**: Results for LOS (median and interquartiles). **b**: Results for TPN (median and interquartiles). Both outcomes were noted to be more than doubled with complex gastroschisis. *Significant difference *P* = < 0.05

There were nine patients (16%) that had a postnatal diagnosis of complex gastroschisis, with an associated longer LOS and duration of TPN (Fig. 1, Table 5). The median LOS for simple gastroschisis (median 30 days, IQR, 23–39) was more than doubled for complex gastroschisis (median 89 days, IQR, 57–147). Similar differences were seen in duration of TPN, with a median 21 days for simple (IQR, 17–31) and 46 days (IQR, 34–187) for complex gastroschisis. Complex gastroschisis babies had the same median gestation at birth of 36.3 weeks (range 33.3–38 weeks) as simple gastroschisis babies median value (range 30–39.3 weeks). The median birth weight was also comparable between complex gastroschisis (2430 g, range 1900–3090 g) and simple gastroschisis (2398 g, range 1300–3860 g).

One woman with an antenatal ultrasound diagnosis of gastroschisis gave birth to a baby with undiagnosed ‘vanishing’ gastroschisis. The baby required surgery for bowel obstruction, malrotation and jejunal atresia, and this complex infant had a lengthy hospital stay of 328 days.

All babies with complex gastroschisis had Australian born mothers except the baby with vanishing gastroschisis whose mother was born in Lebanon. All live-born babies survived to discharge. Follow-up data were available for Australian babies (*n* = 52, 95%) until 1 month after discharge, with no deaths.

## Discussion

We found postnatal diagnosis of complex gastroschisis to be associated with longer LOS and days on TPN, compared to simple gastroschisis, although the median birth weights and gestation at birth were comparable in the two groups. Our study found the only antenatal factor associated with adverse neonatal outcome was multiple dilated bowel loops.

For delivery gestation, a range from 30 weeks to 39^+3^was not associated with longer LOS or greater time on TPN, despite other studies identifying early gestation at birth to impact on outcome [32, 33]. Our findings suggest that concerns relating to short term neonatal outcomes associated with delivery at late preterm gestation may not be as relevant in decision making. Our results could be due to the small number of cases, but other larger studies have found a similar lack of predictive antenatal factors [25, 34].

Stillbirth rates for gastroschisis have improved in high-resource countries from 50% in the 1960s [35] to more recent estimates in research and literature of 4.5–10% [3, 36]. Although there is evidence of an increased risk of intrauterine death [36] with increasing gestation for babies with gastroschisis, we report no late-gestation deaths in our study. Pregnancy surveillance and regular patient education are part of standard care, as is ongoing care from a caseload midwife, and pregnant women are encouraged to contact the hospital or midwife if they are concerned about reduced intrauterine fetal movement. Increased surveillance and patient education leading to earlier delivery of compromised fetuses may be a factor in our study’s absence of late-gestation stillbirths.

Two consistent associations reported with gastroschisis have been young maternal age and smoking; both factors were confirmed in our study. The overall smoking rate in the study group was 27%, which is more than twice the overall smoking rate of 10% in NSW during the same period. It is also an interesting observation in this cohort that in a 5 year period, none of the gastroschisis cases were the result of an assisted reproduction.

Ethnicity has been reported as affecting prevalence, with a higher rate of gastroschisis reported for babies of Caucasian women, and one study reporting a 263% increase in incidence among non-Hispanic black women from the period before 2005 compared to the period 2006–2012 [9]. Our study also revealed ethnicity as a possible factor influencing prevalence. Thirteen percent of our study cohort identified as Aboriginal, which is a higher proportion than that of the NSW population; Aboriginal and Torres Strait Islander women have been recorded as 2.9% of the total NSW female population [37]. In our cohort, non-Australian-born mothers accounted for 27% of babies with gastroschisis (Table 1), which is lower than the NSW average of 35%. For maternal addresses in the Western Sydney Local Health District (WSLHD) subgroup (*n* = 17), 41% of pregnancies with gastroschisis were non-Australian-born (Table 2), in contrast to the high proportion of the culturally and linguistically diverse population of the WSLHD, at over 60% [38]. Of particular note was that none of the pregnancies in this cohort had women born in South Asian countries while they account for 20.8% of all pregnant women delivering in the local health district [38].

Asthma was reported as a pre-existing condition for 34% of women in our study, higher than the 10.9% reported in the NSW female population [39] and may be an area for future investigation.

Increasing awareness of incidence-clustering of gastroschisis has led to calls for further investigation and improved data collection worldwide, to identify possible teratogenic causative agents [40]. The rising prevalence of gastroschisis has been discussed for over 20 years, but there has been no commitment to coordinated data collection including demographic information, environmental assessment, stillbirths, terminations, births and surgical outcomes in Australia. Our study, in a major hospital, was the result of coordination between two hospitals and three departments (maternal fetal medicine specialists at the adult hospital, the children’s hospital neonatal intensive care unit, and the surgical department), and involved piecing together various databases and physical examination of patient records. The Australian and New Zealand Neonatal Network are establishing a surgical network that will improve future surgical neonatal data capture for our region. Ongoing funding, support and commitment to a comprehensive national database of birth anomalies, including collection of data on environmental exposures, ethnicity, terminations and stillbirths, and clinical outcome at tertiary hospitals worldwide, will provide valuable information to inform research into the possible causative agents for the rising prevalence.

Limitations of our study include the retrospective study design, retrospective review of ultrasound data, and small patient numbers. Patient numbers in this study were inadequate to assess the antenatal sonographic predictors for simple versus complex gastroschisis. We suggest further prospective studies into antenatal predictors of complex gastroschisis. However, a strength of our study is that all patients diagnosed with gastroschisis in our region are reviewed by our maternal fetal medicine specialists for counselling, any termination data, including for those terminated at an early gestation, would be captured at our unit.

Conclusions about the causes of rising trends are impossible without robust data collection for all pregnancies, including for all terminations and stillbirths. Improvement in ultrasound techniques and routine early scanning allows for early diagnosis of gastroschisis. A recent study in The Netherlands found that 14% of pregnancies with babies who had isolated gastroschisis were terminated when they were diagnosed before 18 weeks’ gestation [1]. Analysis of five British data repositories from 1991 to 1999 [41] found that 13% of pregnancies with gastroschisis ended in termination. There is no clear reason for no terminations of pregnancies involving babies with gastroschisis in our study. Australia-wide data on reasons for termination and numbers of terminations are unavailable and will continue to confound data on the prevalence of conditions until data are more accurately captured.

All infants born after 25 weeks’ gestation in our cohort survived to discharge in this five-year study period. Improved multidisciplinary care, antenatal surveillance and timely surgical and neonatal care contribute to the current optimal outcomes. Reducing LOS and morbidity is an ongoing objective.

## Conclusion

We found excellent outcomes for late-gestation infants born with gastroschisis, reporting that all live-born infants survived to discharge. In contrast with many other studies, our cohort had no terminations in the study period. In an ethnically diverse population, our finding that there was an increased incidence of infants born with gastroschisis in Australian-born women compared to overseas born women requires more research.

We found no antenatal predictor for hospital LOS, inclusive of gestation at delivery and multiple bowel loop dilation to be associated with greater time on TPN. However complex gastroschisis diagnosed in the postnatal period is associated with greater days on TPN and LOS. This information should be used to counsel parents. The future challenge is to develop consistent specific antenatal ultrasound markers differentiating simple and complex gastroschisis. Better antenatal prediction can provide useful information for parents and families in planning the postnatal period, especially when patients are referred to a tertiary centre from geographically distant areas.

## Abbreviations

CHW: The Children’s Hospital at Westmead
IABD: Intra-abdominal bowel dilatation
LOS: Length of stay
NSW: New South Wales
TPN: Total parental nutrition
WH: Westmead Hospital
WSLHD: Western Sydney Local Health District
